# Molecular Profiling of Advanced Malignancies: A Community Oncology Network Experience and Review of Literature

**DOI:** 10.3389/fmed.2020.00314

**Published:** 2020-07-15

**Authors:** Pritam Tayshetye, Katherine Miller, Dulabh Monga, Candice Brem, Jan F. Silverman, Gene Grant Finley

**Affiliations:** ^1^Department of Hematology-Oncology, Allegheny Health Network, Pittsburgh, PA, United States; ^2^Department of Internal Medicine, Allegheny Health Network, Pittsburgh, PA, United States; ^3^Department of Pathology and Laboratory Medicine, Allegheny Health Network, Pittsburgh, PA, United States

**Keywords:** targeted therapy, genomics, driver mutations, molecular profiling, next generation sequencing, community oncology, precision oncology

## Abstract

**Background:** Many genomic alterations have been identified that are critical to the malignant phenotype. Some of these, termed “driver mutations,” are critical for tumor proliferation and progression. The landscape of targeted therapy has expanded as well. Next-generation sequencing (NGS) of tumors reveals cancer-related genomic alterations and provides therapeutic recommendations for specific targeted therapy. We analyzed our experience with FoundationOne, a validated NGS genomic profiling test, in a community oncology network.

**Methods:** NGS results from May 2014 to September 2016 from a community oncology network in Western Pennsylvania were analyzed. Medical records were reviewed for primary site, stage, biopsy site, time of testing, prior treatment, FDA-approved therapy in patient's and other tumor types and potential clinical trials based upon mutations detected. Two co-primary endpoints for this study were to determine the percentage of patients having mutations with a FDA-approved targeted agent and the percentage of patients in whom a treatment decision was made based on these NGS results.

**Results:** One Fifty-Seven NGS results were available for analysis. 82% patients had a mutation with a FDA-approved targeted agent available while 18% patients had no FDA-approved targeted agent for the mutation detected. Clinical trials were available for 93% cases. The NGS results were utilized in treatment decisions in 18% patients (*n* = 28) with, 7% (*n* = 11) initiating a targeted agent, 6% (*n* = 9) were on an appropriate targeted agent prior to testing and 5% (*n* = 8) being unable to start a targeted agent because of insurance denial, clinical deterioration or patient preference. 38% cases were tested early in the disease course (at diagnosis, during or shortly after first-line treatment) and 62% at progression.

**Conclusions:** NGS is a valuable tool to identify molecular targets for personalizing cancer care. From our experience, the actual number of patients starting a targeted agent based on NGS results is low but it provides substantial information in terms of providing additional treatment options, identifying resistance conferring mutations and facilitating clinical trial enrollment. Optimal time of testing, early or late in disease course, financial implications of testing and using targeted therapy and survival benefit of targeted therapy need further studies.

## Introduction

There are many genomic alterations that have been identified as crucial to the malignant phenotype. Known as “driver” mutations, these alterations have been shown to be vital for tumor formation, proliferation, and progression (1, 2). Chemotherapeutic agents have traditionally been the standard of malignancy treatment, but identification of these driver mutations has led to the development of targeted therapies specifically directed toward these genomic alterations (3–6). With this, the realm of oncology treatment has taken steps toward precision or personalized medicine. While targeted treatments may exhibit little or no superiority over traditional treatments in an unselected population, they have the ability to initiate a dramatic response within a patient population with targetable driver mutations.

The technology for detecting these mutations has advanced as well. Whole genome sequencing (WGS) allows for comprehensive sequencing of whole genomes and whole exomes. Because of the cost, burden of data analysis, and time constraints, WGS is impractical for clinical use, and is limited to a research role. Next Generation Sequencing (NGS), on the other hand, can target a large number of preselected genomic alterations with known significance in malignancy (6, 7). These genomic alterations or molecular targets can be adequately measured in patients' tumors with good quality specimens (8). Subsequently, they can then be used to develop molecular diagnostic protocols, facilitate clinical trial development and to even recommend treatment regimens (9, 10). The implications and utility of NGS testing in clinical practice remain topics of ongoing research. While the pace of development of NGS assays and the output of data from these highly specialized tests continue to increase dramatically, it is important to assess their utility for an oncologist practicing in the community. There are a number of commercially available NGS assays, one of which is FoundationOne. We analyzed our experience with FoundationOne within a community oncology network.

## Materials and Methods

### Study Design

Allegheny Singer Research Institute—West Penn Allegheny Health System Institutional Review Board approval was obtained with waiver of informed consent as the study was a retrospective analysis from case records. NGS results from FoundationOne within an oncology network comprising of academic as well as community cancer centers in seven locations in Western Pennsylvania were retrospectively analyzed from May 2014 through September 2016. The medical records were reviewed for primary site, stage, biopsy site, time of testing, prior treatment, FDA-approved therapy for the patient's and other tumor types, and potential for clinical trials based on the mutations detected through NGS. The time of NGS testing during the patient's course of disease was solely at the discretion of the treating physician which was recorded in the analysis. There were two co-primary endpoints for this study; to determine the percentage of patients having mutations with a FDA-approved targeted agent and the percentage of patients in whom a treatment decision was made based on these NGS results.

### Testing

FoundationOne testing is a comprehensive genomic profiling test which identifies four classes of genomic alterations: base substitutions, insertion and deletion alterations (indels), copy number alterations, and select gene rearrangements. Initially, the assay analyzed 236 genes but currently analyses 324 genes on its FoundationOne CDx platform which requires formalin-fixed paraffin-embedded (FFPE) tissue (11). Blood-based testing for hematological malignancies is done through the FoundationOne Heme platform which sequences DNA of entire coding region of 406 genes and sequences RNA of 265 genes. The results include details about the genomic alterations detected, tumor mutation burden, FDA-approved therapies for the patient's tumor type and other tumor types for the specific genomic alteration as well as potential clinical trials available according to the genomic alteration identified. Further details within the report include a summary of the specific gene and alteration detected and frequency, prognosis and potential treatment strategies for the same.

### Statistics

The study aim was to determine the incidence of patients with targetable mutations and the influence of NGS results on clinical decision making. As such, no statistical hypotheses were examined.

## Results

Overall, there were 157 NGS results available for analysis. Only the NGS results having at least one genomic alteration were included. Only one sample in our study belonging to a patient with chronic myelomonocytic leukemia was from peripheral blood with the rest being FFPE specimens. The tumor types of the 157 results are as shown in Table 1. The median age was 60 years (range 22–90 years) with 77 patients being female and 80 being male. Majority of the cases, 63% (*n* = 99), were stage IV at the time of testing. Stage distribution of all cases is detailed in Table 2.

**Table 1:** Proportion of different tumor types.

**Tumor type**	**Number of samples**
CNS	36 (22.9%)
Lung	23 (14.7%)
Pancreaticobiliary	16 (10.2%)
Genitourinary	15 (9.6%)
Breast	14 (8.9%)
Colorectal	11 (7%)
Unknown primary	7 (4.5%)
Sarcoma	6 (3.8%)
Upper GI	6 (3.8%)
Gynecologic	6 (3.8%)
Others^*^	17 (10.8%)
Total	157 (100%)

* *Includes head & neck (2), thyroid (4), melanoma (4), and one sample each from peripheral blood, appendiceal carcinoma, duodenal carcinoma, hepatocellular carcinoma, non-Hodgkin's lymphoma, desmoid tumor, and parotid tumor*.

**Table 2:** Stage distribution of cases.

**Stage**	**Number of patients**
I	4 (2.5%)
II	5 (3.2%)
III	11 (7%)
IV	99 (63%)
Stage not defined/unstageable tumors (CNS tumors, hematological malignancy, PEComa)	38 (24.2%)

**Figure 1:**
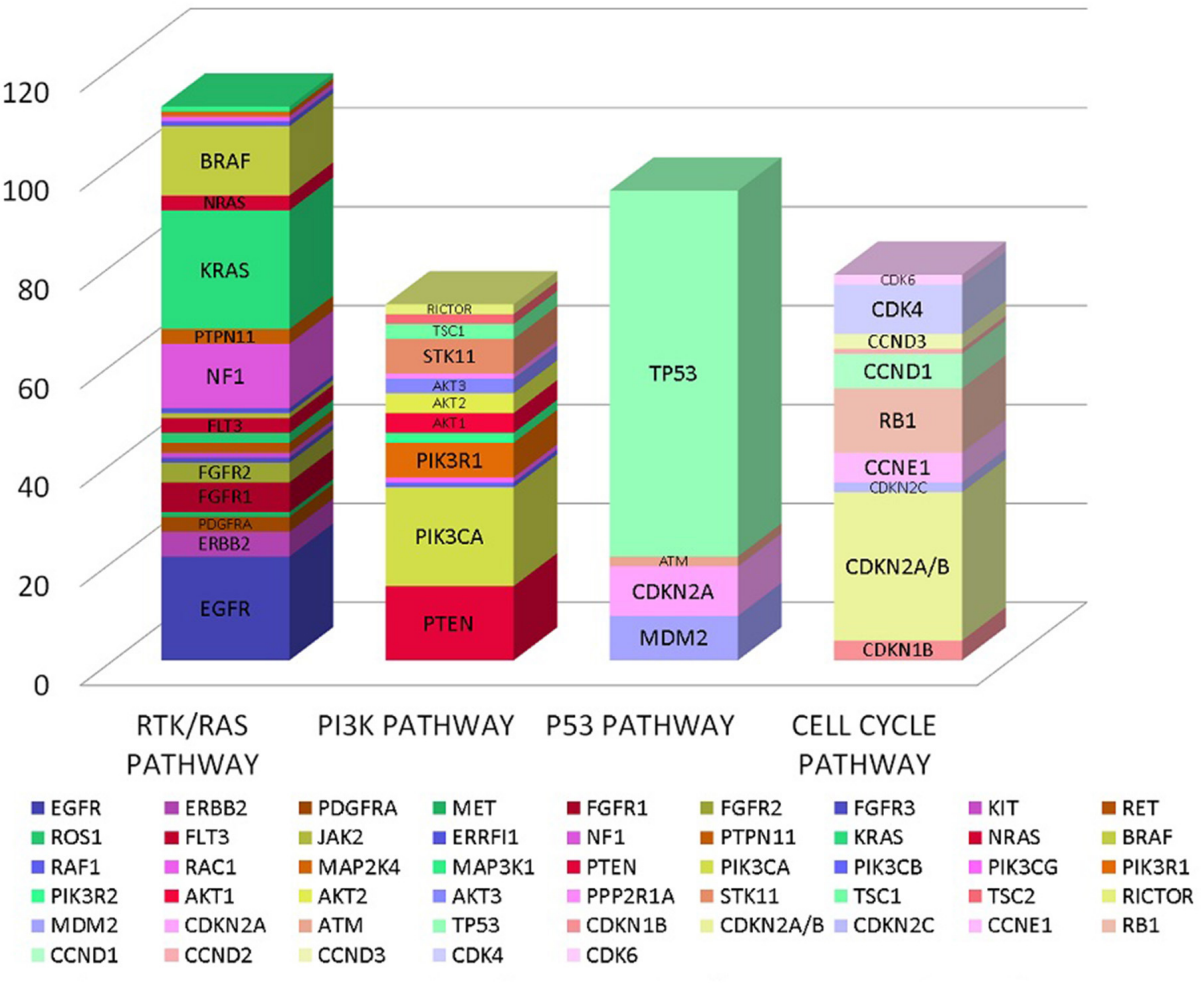
Gene mutation frequencies by oncogenic pathways.

In our analysis we found a total of 185 genes with mutations. These genes encompassed multiple oncogenic signaling pathways in the cancer genome atlas but majority of the mutations occurred in genes associated with RTK/RAS pathway, PI3K pathway, p53 pathway and cell cycle pathway as detailed in Figure 1 (12).

**Figure 2:**
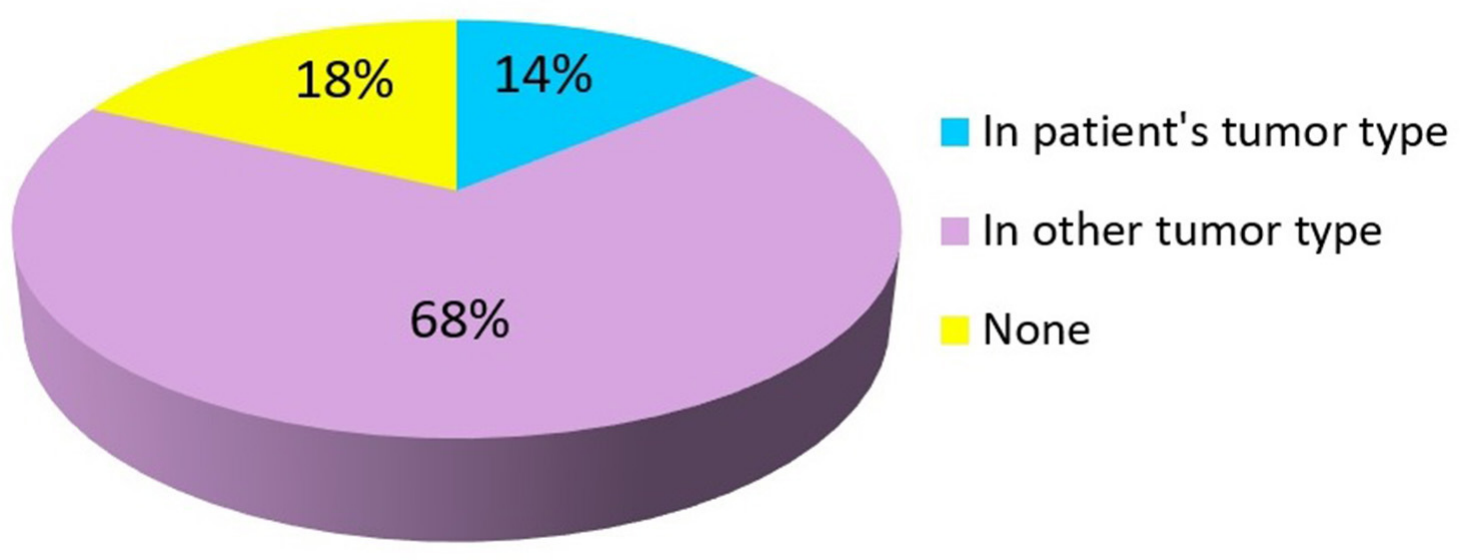
FDA-approved therapies.

Overall, 82% (*n* = 129) patients had a mutation with a FDA-approved targeted agent available while 18% (*n* = 28) patients had no FDA-approved targeted agent for the mutation detected by NGS testing. Of these, FDA-approved targeted agent for a specific mutation present in the patient's primary tumor type was found in 14% (*n* = 22) cases. In 68% (*n* = 107) of cases, FDA-approved targeted agent was found for a specific mutation but with an indication in another tumor type different from the patient's primary tumor as summarized in Figure 2. Clinical trials based on NGS results were available for 93% (*n* = 146) patients.

“Early” testing was defined as NGS testing which was performed at diagnosis, during or shortly after first-line treatment. “Late” testing was defined as NGS testing performed at disease progression. In our study cohort, 38% (*n* = 60) of the patients were tested “early” and 62% (*n* = 97) were tested “late.”

**Figure 3:**
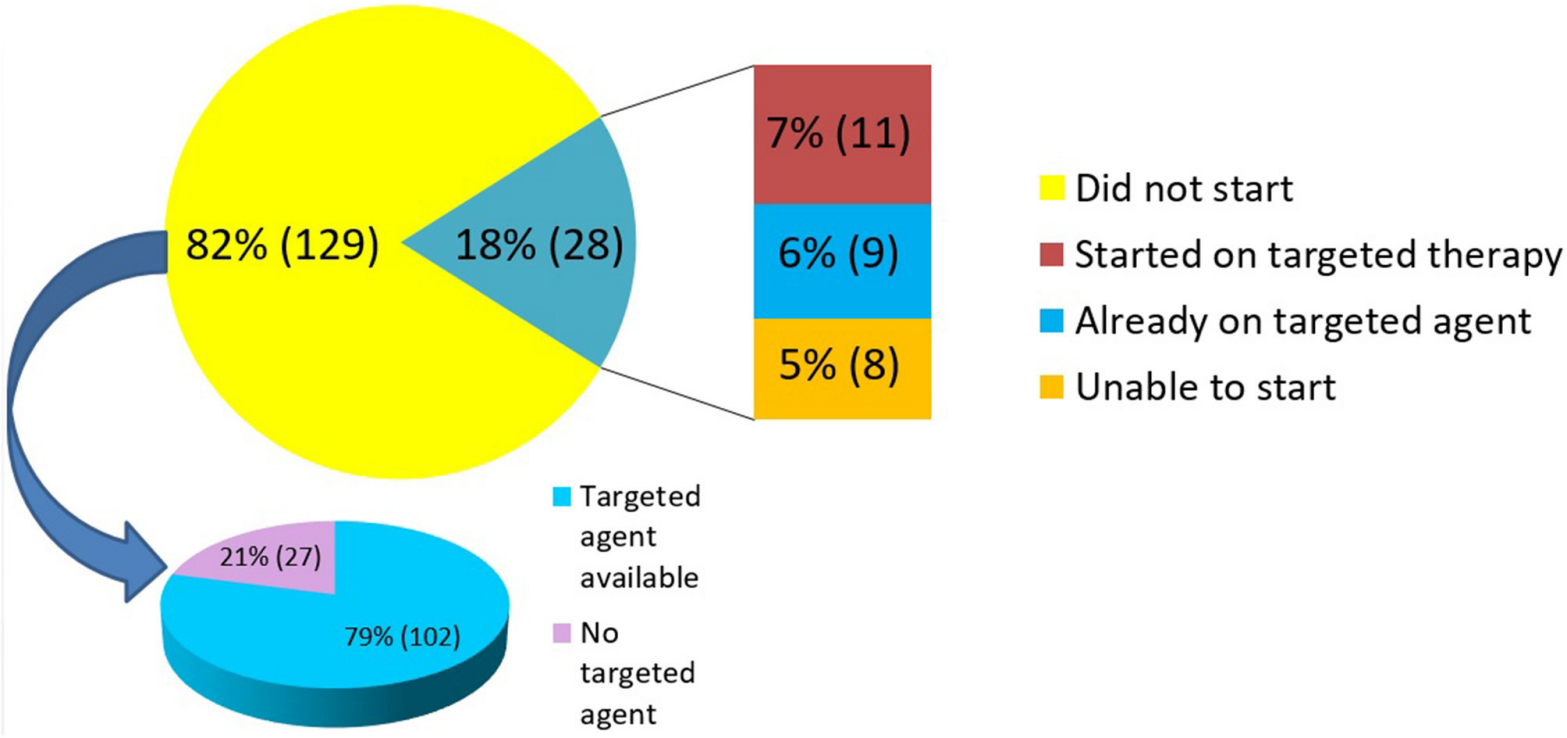
Utilization of NGS results.

NGS results were utilized in treatment decisions in 18% (*n* = 28) of the patients. Of these 28 patients, nine were tested “early” and 19 were tested “late.” 11 of the 28 patients (7% of the entire cohort) were subsequently initiated on a targeted therapy based on their NGS results. The clinical details of these patients are provided in Table 3. The median duration of treatment on targeted therapy was 79 days (range 42–404 days). Nine patients (6% of the entire cohort) were found to be on appropriate targeted agent prior to NGS testing. This cohort included patients in which NGS testing revealed positivity for targets such as HER2/neu (ERBB2) in breast cancer specimens, androgen receptor (AR) in prostate cancer, BRAF in melanoma and EGFR mutation in NSCLC. These patients were on appropriate therapy as the results of these targets were available prior to NGS testing as part of standard practice. The remaining 8 (5% of the entire cohort) were unable to initiate targeted therapy for reasons of insurance denial, clinical deterioration, or patient preference. 82% (*n* = 129) patients did not receive any targeted therapy. Of these, 79% (*n* = 102) had a targeted agent available (in patient's tumor type and/or other tumor type) whereas 21% (*n* = 27) had no therapy option as per NGS testing (Figure 3).

**Table 3:** Clinical parameters of patients on targeted therapy.

**Patient**	**Age**	**Disease site**	**Stage**	**Time of testing**	**Mutations found**	**FDA approved therapies in patient tumor type (with corresponding mutation)**	**FDA approved therapies in another tumor type (with corresponding mutation)**	**Prior lines of therapy**	**Targeted therapy**	**Days on targeted therapy**	**PFS (days)**	**Toxicities**	**Best response**
1	54	Metastatic endometrial adenocarcinoma	IV	Late	NF2, PTEN, ARID1A, CDKN1B, NOTCH2, PIK3R1	None	Everolimus, Temsirolimus (NF2, PTEN)	8	Temsirolimus	63	117	Mucositis grade 2, thrombocytopenia grade 1, rash grade 1, edema grade 1, fatigue grade 2, grade 1 hyperglycemia, grade 2 diarrhea	Decrease in CA-125 from 126.3 U/mL to 79.3 U/mL. No objective response
2	66	Metastatic endometrial adenocarcinoma	IV	Late	PIK3CA, AURKA, EPHB1, GNAS, PPP2R1A, PRKCI	None	Everolimus, Temsirolimus (PIK3CA)	3	Temsirolimus	78	94	Rash grade 1	Decrease in CA-125 from 193.9 U/mL to 148 U/mL. No objective response.
3	43	Metastatic pancreatic adenocarcinoma	IV	Late	BRCA2, KRAS, TP53, SMAD4	None	Olaparib (BRCA2), Cobimetinib (KRAS), Trametinib (KRAS)	4	Olaparib	43	55	Nausea grade 1	No response
4	55	Metastatic cholangiocarcinoma	IV	Late	FGFR2, NF1, CDKN2A/B	None	Pazopanib (FGFR2), Ponatinib (FGFR2), Cobimetinib (NF1), Trametinib (NF1)	2	Pazopanib	79	96	Thrombocytopenia grade 1, hypertension grade 3	No response
5	73	Glioblastoma multiforme	-	Early	NF1, CDKN2A/B, CHD2, PTPN11, TERC, TERT	None	Cobimetinib (NF1), Everolimus (NF1), Temsirolimus (NF1), Trametinib (NF1)	Surgery + chemoradiation with Temozolomide	Everolimus, Bevacizumab, Optune device	333	298	Decline in pulmonary function testing grade 2	Radiographically stable disease
6	47	Glioblastoma multiforme	-	Late	PDGFRA, CDKN2A/B, CDKN2C, TERT	None	Dasatinib (PDGFRA), Everolimus (PDGFRA), Imatinib (PDGFRA), Nilotinib (PDGFRA), Pazopanib (PDGFRA), Ponatinib (PDGFRA), Regorafenib (PDGFRA), Sorafenib (PDGFRA), Sunitinib (PDGFRA), Temsirolimus (PDGFRA)	Surgery + chemoradiation with Temozolomide + 3 lines of chemotherapy	Imatinib, Bevacizumab, Temozolomide	110	110	Thrombocytopenia grade 4	No response
7	50	Metastatic papillary thyroid carcinoma	IV	Late	RET, NF2, STK11, RBM10, SMAD4, TERT	Lenvatinib (RET), Sorafenib (RET)	Cabozantinib (RET), Ponatinib (RET), Regorafenib (RET), Sunitinib (RET), Vandetinib (RET), Everolimus (NF2,STK11), Temsirolimus (NF2,STK11) Lapatinib (NF2), Trametinib (NF2)	3	Lenvatinib	100	100	Hematuria grade 2, thrombocytopenia grade 2	No response
8	88	Lung adenocarcinoma + synchronous metastatic renal cell carcinoma	IB + IV	Early	BRAF, PIK3CA, SETD2, SMAD4	None	Dabrafenib (BRAF), Regorafenib (BRAF), Trametinib (BRAF), Vemurafenib (BRAF), Everolimus (PIK3CA), Temsirolimus (PIK3CA)	None	Everolimus	68	68	Pneumonitis grade 3	No response
9	47	Metastatic breast cancer	IV	Late	PIK3CA, CCND1, CDH1, EMSY, FGF19, FGF3, FGF4	Everolimus (PIK3CA)	Temsirolimus (PIK3CA)	3	Everolimus	127	102	Rash grade 1, fatigue grade 1	No response
10	68	Metastatic pancreatic adenocarcinoma	IV	Late	STK11, ARID2, DNMT3A, FLCN, TERT, U2AF1	None	Everolimus (STK11), Temsirolimus (STK11)	2	Everolimus	42	59	Nausea grade 1, fatigue grade 2, epistaxis grade 1, myalgia grade 2	No response
11	67	Metastatic adenoid cystic carcinoma	IV	Late	PIK3CA, PIK3R1, CREBBP, MYB	None	Everolimus (PIK3CA, PIK3R1), Temsirolimus (PIK3CA, PIK3R1)	2	Everolimus	404	389	Dizziness grade 3	Radiographically stable disease

## Discussion

In our retrospective analysis of NGS testing of 157 patients from May 2014 through September 2016 we found that targeted therapies and clinical trials were available in 82% and 93% of the patients, respectively. However, treatment decisions based on these NGS results were utilized in only 18% of the patients with only 7% of the patients initiating NGS directed therapy. In 5% of patients where the patients were unable to start therapy due to clinical deterioration, insurance denial or patient preference, the treating physicians' decision to utilize results of NGS testing and initiate NGS directed therapy was acknowledged. Our analysis revealed that majority of the patients (79%) did not initiate targeted therapy despite having a targeted agent as specified by NGS testing. The most common reason for not utilizing the NGS results was unavailability of an FDA-approved targeted agent in the tumor type which was being treated and availability of an FDA-approved standard of care regimen. Opting for a non-approved targeted therapy over standard of care thus contributed to the clinician's dilemma. Insurance approval for an off-label indication of a targeted drug when other standard of care therapies are available is a major challenge as well. In this situation, a multidisciplinary recommendation from a specialty molecular tumor board would potentially be beneficial to aid clinician's decision-making, which unfortunately was unavailable during the study period. Also, in minority of cases, clinical deterioration of the patient precluded any further therapy. While the actual number of patients initiating a targeted agent based on NGS results was low based on our experience, it is comparable to other studies. Johnson et al. analyzed 103 patients, identifying potentially actionable genetic alterations in 83% of tumors with 21% of these patients receiving genotype directed therapy (13). The ProfiLER molecular profiling trial identified actionable alterations in 51% and treatment recommendation in 35% in analysis of 2490 patients with ultimately only 7% initiating the recommended therapy (14).

The outcomes of molecularly matched therapy have been explored by a few trials. In 2010, Van Hoff et al. compared progression free survival (PFS) between regimens selected by molecular profiling with the most recent regimen on which the patient had experienced progression and showed a longer PFS on a regimen suggested by molecular profiling. Ascertainment bias, PFS ratio as an endpoint and non-randomized nature of the study were important issues highlighted by the authors (8). MOSCATO 01 trial was also a prospective trial evaluating PFS benefit of genomics matched therapy compared to prior therapy in hard-to-treat advanced cancers. Their results indicated improved outcomes in only a subset of patients with only 7% of the screened patients benefiting from this approach (15). The SHIVA trial was a phase two trial which did not report any PFS benefit of molecularly targeted agents outside their indications in heavily pretreated patients with metastatic cancer (16). In comparison, the Princess Margaret IMPACT/COMPACT trial showed a higher overall response rate in patients treated on genotype-matched clinical trials compared with genotype-unmatched trials (17). The EXACT trial conducted in Austria showed a better PFS in 34 out of 55 patients (112 vs. 61 days) with molecularly targeted therapy compared to the previous treatment (18). In our study, the PFS analysis was not feasible because of small number of patients with different malignancies on NGS suggested targeted therapies and relative short mean treatment duration of 79 days. Of the patients initiated on a genomics matched therapy, majority had no response to therapy and all had at least grade 1 toxicity as detailed in Table 3.

While some driver mutations are targetable, others have shown to confer resistance to standard therapy choices such as KRAS, BRAF, and PIK3CA mutations in colorectal cancer (19–21). The optimal time of testing, whether early in the disease course vs. late in the disease course, remains a point of contention. Earlier testing has the potential to identify resistance conferring mutations and direct patients away from a futile therapy such as EGFR directed therapy for KRAS mutations in colorectal tumors. It also has the possibility to allow for earlier enrollment in clinical trials, with the potential for better response rates. The I-PREDICT (Investigation of Profile-Related Evidence Determining Individualized Cancer Therapy) trial of 47 patients found high molecular matching rates of 36%, high rates of stable disease >6 months/complete response/partial response at 53%, and improved PFS in treatment naïve tumors in patients with metastatic and/or unresectable, untreated lethal cancers on targeted therapies determined by NGS testing (22). Some patients in our study had potential treatment options available but could not be started on a targeted agent because of clinical deterioration. Whether NGS testing earlier in disease course could have influenced the clinical course of these patients remains an interesting question. On the other hand, financial implications of testing earlier when standard of care options are available, need to be considered as well.

While there are numerous potential benefits from the use of NGS, further studies are still needed to determine its full clinical utility (9). Recognition of targetable mutations through NGS testing allows for expansion of treatment options, identification of resistance conferring mutations, and the ability to pinpoint clinical trials for enrollment. To harness the true potential of NGS testing, National Cancer Institute (NCI) and American Society of Clinical Oncology (ASCO) have started enrolling patients in “basket” clinical trials or single arm sub-studies to identify signals of clinical activity. Two of the larger trials in this setting are the NCI-MATCH (molecular analysis for therapy choice) trial and ASCO TAPUR (targeted agent and profiling utilization registry) study^1^.

As molecular tumor profiling continues to evolve and expand, the number of targeted agents is certain to increase, along with the number of clinical trials available for patients (6, 10, 13). There are several national initiatives underway which seek to harmonize clinical and molecular data from cancer patients. These include the American Association for Cancer Research's Project GENIE (Genomics Evidence Neoplasia Information Exchange), the NCI's Genomic Data Commons, ASCO's CancerLinQ project, which will expand public access to de-identified clinical and genomic data. These databases will aggregate data from a global community of participating organizations, including healthcare providers, research institutions, life sciences companies, government agencies, and individual researchers. In the event of geographical, institutional or other limitations to patient participation in relevant trials, expanded genomic data projects can provide the clinician with more personalized guidance on treatment selection based on data from patients with similar characteristics. This will ultimately improve the evidence-based quality of care that the patients will receive (23–25).

With the recent FDA approval of FoundationOne CDx NGS, expanded coverage of the testing will lead to its increased utilization^2^. The output of data by these targeted panel sequencing tests is increasing exponentially as well. As described by Horak et al., the cost of NGS testing continues to decrease making genomic analysis of tumors a common practice going forward adding a layer of complexity to decision making for clinicians. The confidence to interpret these complex tests and use them for clinical decision making in daily practice can be daunting for clinicians in general, especially for those in community practice. It is a challenge for the oncology community to generate high-quality NGS data and integrate them with histopathological and clinical findings followed by appropriate clinical trials (26). One step in this direction is incorporation of molecular tumor boards with faculty well-versed with molecular testing to assist clinicians in interpreting complex NGS reports, guiding treatment decisions and designing clinical trials. Distinction between a “driver” mutation and “passenger” mutation could be perplexing, wherein a multidisciplinary expert discussion at a molecular tumor board could be helpful. Many institutions, including ours, have implemented molecular tumor board discussions as a part of routine practice.

There are few limitations to our study. Firstly, the study is a retrospective analysis of NGS results over a period of more than 2 years. Secondly, multiple tumor types were analyzed with relatively small sample sizes. Thirdly, the FoundationOne testing also evolved over that time period with inclusion of 324 genes compared to the initial 236. Since most of our patients were from a community practice network, majority of the clinical trials utilizing targeted therapy were unavailable locally.

Recently, the Right to Try bill was signed into law. It seeks to grant all terminally ill patients access to experimental therapies once approved alternatives have failed. ASCO released a position statement in April 2017 wherein although increased access to investigational drugs is supported, it is highlighted that the potential safety and efficacy of investigational drugs that is monitored by independent review is bypassed in these laws, exposing patients to a potentially toxic drug without clearly establishing efficacy. Two other equally important factors that these laws do not address are the obligations for manufacturers to provide access to the investigational product, and for insurers to provide coverage for the cost of the drug and the potential complications of therapy. In addition, access to the experimental therapies as a part of Right to Try could potentially limit clinical trial enrollment (27–29).

The financial implications of NGS testing are also of importance, especially as more attention is directed toward the cost of healthcare, and payment models. Additionally, long term studies analyzing the survival benefit of targeted therapy should also be pursued. Better response rates, whether in combination with chemotherapy, or alone, may provide longer and better quality of life for patients, lead to newer innovations, and reduce costs in the long run. Other future directions include the creations of algorithms to identify optimal time for testing as well as the selection of appropriate patients to test. Strategies to improve the financial feasibility of testing for patients should also be pursued.

## Conclusion

In our genomic profiling analysis of 157 patients from a community oncology network in Western Pennsylvania, we found that 82% patients had a mutation which had a FDA-approved targeted agent available. Thus, our study provides proof of concept that NGS is a valuable tool to identify molecular targets for personalizing cancer care. The gain is substantial in terms of providing further treatment options for patients, identifying resistance conferring mutations and clinical trials that will provide the most benefit for the patient. Although the number of patients initiating treatment with a targeted agent based on NGS results is currently low, this number is likely to rise as clinician comfort with interpretation of results improves with incorporation of molecular tumor boards and expanded insurer coverage with the advent of FDA approval of FoundationOne CDx testing. Further studies are still needed to determine full clinical utility of NGS, especially regarding time of testing and survival benefit of targeted therapy. Increase in the number of targetable mutations, expansion of clinical trials, and the push by the greater oncology community to create genomic databases are also likely to drive an increase in NGS utilization in clinical practice in this era of precision medicine.

## Data Availability Statement

The datasets generated for this study are available on request to the corresponding author.

## Author Contributions

PT: data extraction and analysis and manuscript drafting. KM, DM, and GF: manuscript editing. CB and JS: obtaining test results. All authors contributed to the article and approved the submitted version.

## Conflict of Interest

The authors declare that the research was conducted in the absence of any commercial or financial relationships that could be construed as a potential conflict of interest.

## Footnotes

^1^Available online at: https://ecog-acrin.org/trials/nci-match-eay131, Available online at: https://www.tapur.org/

^2^Available online at: https://www.accessdata.fda.gov/cdrh_docs/pdf17/P170019a.pdf

## References

[1] Levine AJ Oren M. The first 30 years of p53: Growing ever more complex. Nat Rev Cancer. (2009) 9:749–58. 10.1038/nrc2723 19776744 PMC2771725

[2] Bos JL. ras oncogenes in human cancer: a review. Cancer Res. (1989) 49:4682–9. 2547513

[3] Bergethon K Shaw AT Ou SH Katayama R Lovly CM McDonald NT. ROS1 rearrangements define a unique molecular class of lung cancers. J Clin Oncol. (2012) 30:863–70. 10.1200/JCO.2011.35.6345 22215748 PMC3295572

[4] Soda M Choi YL Enomoto M Takada S Yamashita Y Ishikawa S. Identification of the transforming EML4-ALK fusion gene in non-small-cell lung cancer. Nature. (2007) 448:561–6. 10.1038/nature05945 17625570

[5] Kris MG Johnson BE Berry LD Kwiatkowski DJ Iafrate AJ Wistuba II. Using multiplexed assays of oncogenic drivers in lung cancers to select targeted drugs. JAMA. (2014) 311:1998–2006. 10.1001/jama.2014.3741 24846037 PMC4163053

[6] Dias-Santagata D Akhavanfard S David SS Vernovsky K Kuhlmann G Boisvert SL. Rapid targeted mutational analysis of human tumors: a clinical platform to guide personalized cancer medicine. EMBO Mol Med. (2010) 2:146–58. 10.1002/emmm.201000070 20432502 PMC3377316

[7] Biesecker LG Green RC. Diagnostic clinical genome and exome sequencing. N Engl J Med. (2014) 370:2418–25. 10.1056/NEJMra1312543 24941179

[8] Von Hoff DD Stephenson JJ Rosen P Loesch DM Borad MJ Anthony S. Pilot study using molecular profiling of patients' tumors to find potential targets and select treatments for their refractory cancers. J Clin Oncol. (2010) 28:4877–83. 10.1200/JCO.2009.26.5983 20921468

[9] Khoury MJ McBride CM Schully SD Ioannidis JP Feero WG Janssens AC. The scientific foundation for personal genomics: recommendations from a national institutes of health-centers for disease control and prevention multidisciplinary workshop. Genet Med. (2009) 11:559–67. 10.1097/GIM.0b013e3181b13a6c 19617843 PMC2936269

[10] Servant N Roméjon J Gestraud P La Rosa P Lucotte G Lair S. Bioinformatics for precision medicine in oncology: principles and application to the SHIVA clinical trial. Front Genet. (2014) 5:1–16. 10.3389/fgene.2014.00152 24910641 PMC4039073

[11] Available, online at: https://assets.ctfassets.net/vhribv12lmne/6Rt6csmCPuaguuqmgi2iY8/e3a9b0456ed71a55d2e4480374695d95/FoundationOne_CDx.pdf

[12] Sanchez-Vega F Mina M Armenia J Chatila WK Luna A La KC. Oncogenic signaling pathways in the cancer genome atlas. Cell. (2018) 173:321–37.e10. 10.1016/j.cell.2018.03.035 29625050 PMC6070353

[13] Johnson DB Dahlman KH Knol J Gilbert J Puzanov I Means-Powell J Enabling a genetically informed approach to cancer medicine: a retrospective evaluation of the impact of comprehensive tumor profiling using a targeted next-generation sequencing panel. Oncologist. (2014) 19:616–22. 10.1634/theoncologist.2014-0011 24797823 PMC4041676

[14] Tredan O Corset V Wang Q Varnier R Pacaud C Torroja A Routine molecular screening of advanced refractory cancer patients: an analysis of the first 2490 patients of the ProfiLER study. J Clin Oncol. (2017) 35 (suppl):abstr LBA100). 10.1200/JCO.2017.35.18_suppl.LBA100

[15] Massard C Michiels S Ferté C Le Deley MC Lacroix L Hollebecque A. High-throughput genomics and clinical outcome in hard-to-treat advanced cancers: results of the MOSCATO 01 trial. Cancer Discov. (2017) 7:586–95. 10.1158/2159-8290.CD-16-1396 28365644

[16] Le Tourneau C Delord JP Gonçalves A Gavoille C Dubot C Isambert N Molecularly targeted therapy based on tumour molecular profiling versus conventional therapy for advanced cancer (SHIVA): a multicentre, open-label, proof-of-concept, randomised, controlled phase 2 trial. Lancet Oncol. (2015) 16:1324–34. 10.1016/S1470-2045(15)00188-6 26342236

[17] Stockley TL Oza AM Berman HK Leighl NB Knox JJ Shepherd FA. Molecular profiling of advanced solid tumors and patient outcomes with genotype-matched clinical trials: the Princess Margaret IMPACT/COMPACT trial. Genome Med. (2016) 8:109. 10.1186/s13073-016-0364-2 27782854 PMC5078968

[18] Prager GW Unseld M Waneck F Mader R Wrba F Raderer M Results of the extended analysis for cancer treatment (EXACT) trial: a prospective translational study evaluating individualized treatment regimens in oncology. Oncotarget. (2019) 10:942–52. 10.18632/oncotarget.26604 30847023 PMC6398177

[19] Karapetis CS Khambata-Ford S Jonker DJ Callaghan CJ Tu D. K-ras mutations and benefit from cetuximab in advanced colorectal cancer. N Engl J Med. (2008) 359:1757. 10.1056/NEJMoa0804385 18946061

[20] Pietrantonio F Petrelli F Coinu A Di Bartolomeo M Borgonovo K Maggi C. Predictive role of BRAF mutations in patients with advanced colorectal cancer receiving cetuximab and panitumumab: a meta-analysis. Eur J Cancer. (2015) 51:587–94. 10.1016/j.ejca.2015.01.054 25673558

[21] Mao C Yang ZY Hu XF Chen Q Tang JL. PIK3CA exon 20 mutations as a potential biomarker for resistance to anti-EGFR monoclonal antibodies in KRAS wild-type metastatic colorectal cancer: a systematic review and meta-analysis. Ann Oncol. (2012) 23:1518. 10.1093/annonc/mdr464 22039088

[22] Sicklick JK Leyland-Jones B Kato S Hahn M Williams C De P Personalized, molecularly matched combination therapies for treatment-na. J Clin Oncol. (2017) 35:15_suppl:2512-2512. 10.1200/JCO.2017.35.15_suppl.2512

[23] American, Association for Cancer Research Project Genomics Evidence Neoplasia Information Exchange (GENIE), Available online at: https://www.aacr.org/Research/Research/Pages/aacr-project-genie.aspx

[24] NCI, Genomic Data Commons, Available online at: https://gdc.cancer.gov/

[25] American Society of Clinical Oncology CancerLinQ Available online at: https://cancerlinq.org/

[26] Horak P Fröhling S Glimm H. Integrating next generation sequencing into clinical oncology: strategies,promises and pitfalls. ESMO Open. (2016) 1:e000094. 10.1136/esmoopen-2016-000094 27933214 PMC5133384

[27] American Society of Clinical Oncology Position Statement On Access to Investigational Drugs Available online at: https://www.asco.org/sites/new-www.asco.org/files/content-files/blog-release/documents/2017-Access-to-Investigational-Drugs-Position-Statement.pdf?et_cid=39110097&et_rid=1760459169&linkid=full+statement

[28] American Society of Clinical Oncology ASCO in Action Brief: Right-to-Try and Expanded Access to Investigational Drugs. (2018). Available online at: https://www.asco.org/advocacy-policy/asco-in-action/asco-action-brief-right-try-and-expanded-access-investigational-drugs

[29] Sigal Ellen V Why Right-to-Try Laws Are Dangerous. The ASCO Post. (2018). Available online at: http://www.ascopost.com/issues/march-5-2018-special-report/why-right-to-try-laws-are-dangerous/

